# The Vertebrate Genome Annotation browser 10 years on

**DOI:** 10.1093/nar/gkt1241

**Published:** 2013-12-06

**Authors:** Jennifer L. Harrow, Charles A. Steward, Adam Frankish, James G. Gilbert, Jose M. Gonzalez, Jane E. Loveland, Jonathan Mudge, Dan Sheppard, Mark Thomas, Stephen Trevanion, Laurens G. Wilming

**Affiliations:** Wellcome Trust Sanger Institute, Wellcome Trust Genome Campus, Hinxton, Cambridgeshire CB10 1HH, UK

## Abstract

The Vertebrate Genome Annotation (VEGA) database (http://vega.sanger.ac.uk), initially designed as a community resource for browsing manual annotation of the human genome project, now contains five reference genomes (human, mouse, zebrafish, pig and rat). Its introduction pages have been redesigned to enable the user to easily navigate between whole genomes and smaller multi-species haplotypic regions of interest such as the major histocompatibility complex. The VEGA browser is unique in that annotation is updated via the Human And Vertebrate Analysis aNd Annotation (HAVANA) update track every 2 weeks, allowing single gene updates to be made publicly available to the research community quickly. The user can now access different haplotypic subregions more easily, such as those from the non-obese diabetic mouse, and display them in a more intuitive way using the comparative tools. We also highlight how the user can browse manually annotated updated patches from the Genome Reference Consortium (GRC).

## INTRODUCTION

In 2014, the Vertebrate Genome Annotation (VEGA) (http://vega.sanger.ac.uk) browser will celebrate its 10th anniversary. It was initially designed as a community resource for browsing manual annotation, produced by the Human And Vertebrate Analysis aNd Annotation (HAVANA) team based at the Wellcome Trust Sanger Institute (WTSI), of finished sequence from the Human Genome Project (HGP) (1). At its launch VEGA contained only 10 finished chromosomes from the human genome and a few small genomic regions from mouse and zebrafish (2). It was thought that the manual annotation may not be needed past the completion of the human reference genome and that automated gene builds provided by Ensembl may be sufficient for the researchers needs. However, with the launch of the Encyclopedia of DNA Elements (ENCODE) (3) project in 2004, it was recognized that a combination of manual and automated annotation was the optimum way to annotate the human genome. Therefore, as part of the GENCODE project (4), manual annotation, and a tool for viewing it, persisted.

The VEGA website runs from an Ensembl (5) schema database and is kept synchronized with that of the Ensembl website. This strategy has the advantage that when new features are developed for Ensembl they can become available to VEGA with little or no development time being required. In terms of the annotation data themselves, for the primary species (human, mouse, zebrafish and pig) they are presented first in VEGA and then in Ensembl, both as distinct gene sets in the browser itself and also as part of the Ensembl merged gene set. Since this requires projecting the annotation between assemblies without changing it, to maximize the amount of annotation that can be viewed in this way, we keep, wherever possible, the genome reference sequence versions the same in the two browsers. Assemblies can be different within the two browsers, since the HAVANA team annotates sequence updates and haplotypes before they have been released by the Genome Reference Consortium (GRC) (http://www.ncbi.nlm.nih.gov/projects/genome/assembly/grc/). This close partnership with Ensembl allows us to display community annotation, such as that from the pig immune response annotation group (6), in Ensembl and VEGA and also enables its future merge into Ensembl’s automatic gene builds.

**Figure 1.**
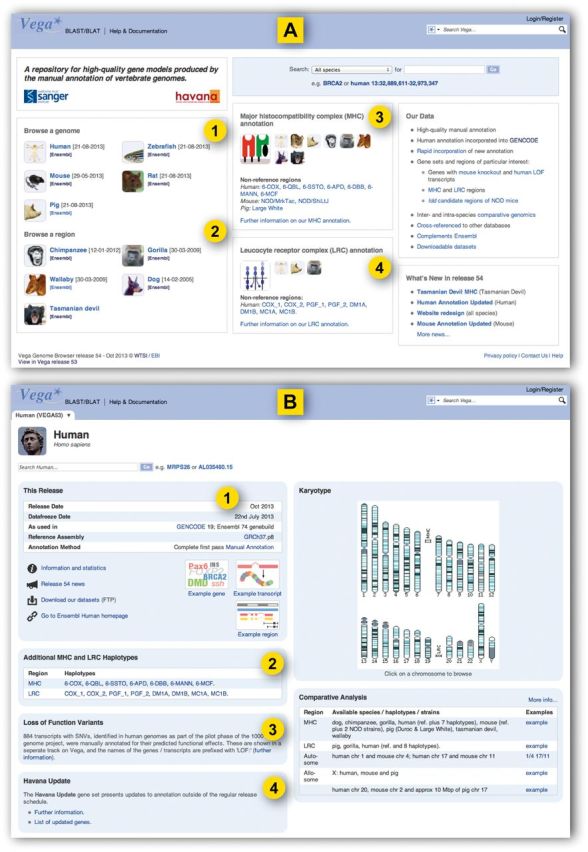
Redesigned VEGA home page and species landing pages. **(A)** New home page with complete genomes (1) separated from partial regions (2), and a new panel with alternative entry points to special data sets available in multiple genomes (3, 4). **(B)** New species landing page; human shown here. Easy access to statistics and examples (1), special data sets (2, 3) and updated annotation (4).

To enable users to navigate the different datasets easily, the VEGA introduction pages have been redesigned (see Figure 1) to highlight the difference between whole genome datasets and partial regions. Currently, VEGA has five reference genomes—human, mouse, zebrafish, pig and rat—which are the main focus of manual annotation by the HAVANA team. Uniquely, VEGA also has small regions from other species that are important for comparative analysis of specific gene families, such as immunoglobulins, or regions of medical importance, such as the major histocompatibility complex (MHC) (7). Historically, the HAVANA group has had a special interest in analysing genomic regions containing MHC and leukocyte receptor complex (LRC) (7) gene clusters because of sequence generated for these by the WTSI. MHC and LRC regions have been sequenced and annotated in eight different human haplotypes. The MHC is of medical interest because it has been linked to many genetic determinants for autoimmune diseases and to some infectious diseases (7). It contains many immune related genes, including highly polymorphic genes encoding MHC class I and class II molecules that present antigens to T lymphocytes. The MHC region has also been annotated in mouse (three strains), gorilla (8), chimpanzee, wallaby (9), Tasmanian devil (10) and pig (11), the latter in two haplotypes. All, except the chimpanzee genomic sequence, have been sequenced *de novo* using clone-based techniques (or whole genome shotgun for pig reference); the chimpanzee sequence has been previously sequenced and published by Anzai *et al.* (12).

## ANNOTATION BIOTYPES AND STATISTICS

Since the first release of VEGA in 2004, the classifications of loci and their transcripts have increased considerably in complexity. Our aim with the classification of loci and transcripts into different biotypes is to confer to the user functionality and confidence information. Originally there were only four gene statuses—known, novel, putative and predicted—which described the level of confidence an annotator had in the annotation. This was used in combination with the following gene biotypes: protein-coding gene, pseudogene or processed transcript. Because of the complexity of transcription within loci, as well as the desire to have more fine-grained classifications, we now have an expanded list of gene biotypes (Table 1).

**Table 1.** Biotypes available in VEGA, with a brief description of each

Biotype	Description
Protein coding	
Polymorphic	At least one variant has a valid ORF and at least one coding variant contains a polymorphism (see ‘NOVEL GENE TRACKS IN VEGA: LOF AND KO’ section).
Protein coding (in progress)	‘Zebrafish only’. Genome assembly issue causes loss of ORF; to be re-annotated on correct assembly.
lncRNA	Long non-coding RNA: lacks protein-coding potential and is >200 bp long.
Non-coding	Known from publications to be non-coding.
3-Prime overlapping	Transcriptional start site and/or published experimental data support independent transcription from the 3′ UTR of a coding gene.
Antisense	At least one variant overlaps a protein-coding locus on the opposite strand, or antisense regulation of a coding gene has been published.
lincRNA	Long intergenic ncRNA: does not overlap (sense nor antisense) a coding gene.
Sense intronic	In an intron of a coding gene; no exonic overlap.
Sense overlapping	Contains a coding gene in an intron; no exonic overlap.
Pseudogene	ORF disrupted by frameshifts and/or premature stop codons.
Processed	Lacks introns and arose from retrotransposition of parent gene mRNA.
Unprocessed	Can contain introns and is produced by genomic duplication.
Transcribed	Locus-specific transcripts indicate transcription. These can be classified into ‘Processed’, ‘Unprocessed’ and ‘Unitary’.
Translated	Locus-specific protein mass spectroscopy data suggests translation. These can be classified into ‘Processed’ and ‘Unprocessed’. We maintain the connection with the pseudogene biotype until the experimental community validates it as a coding gene.
Polymorphic	Pseudogene owing to a SNP/DIP, but orthologous gene translated in other individuals/haplotypes/strains.
Unitary	Species-specific unprocessed pseudogene without a parent gene, which has an active orthologue in another species.
IG	Immunoglobulin pseudogene.
IG Gene	Immunoglobulin gene.
TR Gene	T-cell receptor gene.

The largest change has been the annotation of long non-coding genes, which were classified simply as ‘processed transcripts’ in 2004. In VEGA we currently have more than 13 000 lncRNA genes annotated on the human reference genome, the majority of which are classified as long intergenic non-coding RNAs (lincRNAs). Recent publications using publicly available RNA-seq datasets predict that the number of lncRNAs identified on the human genome could exceed that of the protein-coding genes (13). In addition, the number of annotated pseudogenes has increased and recent publications demonstrate that ∼20% of pseudogenes in human show evidence of transcription (using EST and RNA-seq alignments) (14). A much smaller fraction, <1%, could yield a new translation product, as indicated by shotgun proteomic experiments in mouse (15).

We have recently reclassified readthrough transcripts as separate loci, where previously they were often annotated as a splice variant of one of the loci linked by the readthrough transcript. Classifying readthrough transcripts separately makes them easier to identify and to filter out if necessary. Readthroughs tend to confound automatic prediction algorithms and their tagging will help refine automatic annotation pipelines. The readthrough reclassification was instituted in agreement with RefSeq at NCBI, whom we collaborate closely with on the consensus coding sequence (CCDS) dataset (16). Whether or how many readthrough transcripts are functional is yet to be determined; targeted mass spectrometry is being used to identify and validate some readthrough RNAs (17).

**Figure 2.**
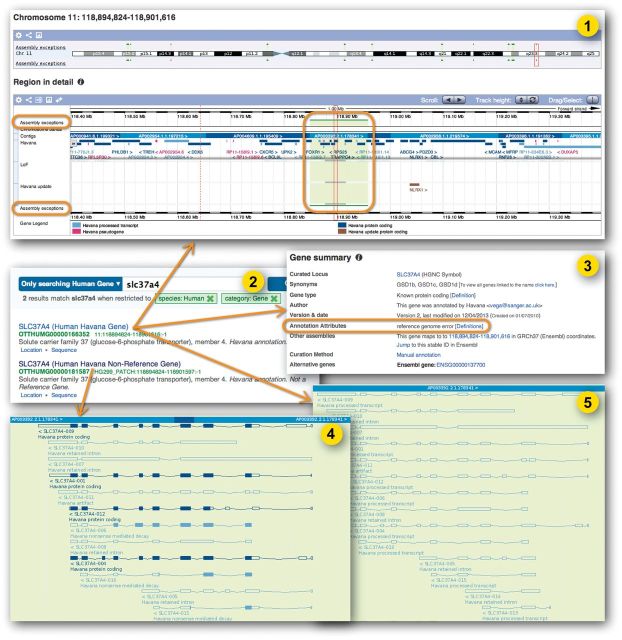
Viewing patches in VEGA. Searching VEGA for the human SLC37A4 gene yields two results (panel 2): one hit on the reference genome (top) and one on a patch (bottom). The top of the ‘Location’ page (panel 1) for the reference gene shows the location of patches on the chromosome, with the region shown in detail boxed in red. The detail view panel shows the location of the patch as two green lines in the ‘Assembly exceptions’ track (highlighted with an orange box left) and light green shading between them (highlighted with an orange box middle). The ‘Gene’ page (panel 3) for the reference gene shows the remark ‘reference genome error’ under the ‘Annotation Attributes’ section of the ‘Gene summary’ (highlighted with an orange box). Panels 4 and 5 show the difference in annotation of the same gene on the patch and reference, respectively. Note the lack of any CDS annotation on the reference gene.

The gene statistics for each genome have changed in the new release, VEGA 53, to give a more comprehensive and fine-grained overview of the gene biotypes annotated on a given genome. The statistics take into account genes annotated on the patch sequences the GRC provide. Human and mouse genome assemblies are updated regularly by the GRC through the issuing of alternate sequences in the form of ‘fix’ patches (which correct sequence errors or fill gaps) and ‘novel’ patches (which correct assemblies or fill gaps) (18). Patches are manually annotated and the corrected genes can be viewed alongside the current assembly. One example of a ‘fix’ patch is HG79_PATCH on human chromosome 9. It corrects the ABO gene, which, in the reference GRCh37 genome, locates to two clones that originated from two different haplotypes and does not code in that artificial configuration. Genes that do not code because of genome sequence or assembly errors are given a ‘reference genome error’ attribute, which is visible on the VEGA Gene page under ‘Annotation Attributes’. The current list of standardized annotation attributes are defined on the VEGA info pages (http://vega.sanger.ac.uk/info/about/annotation_attributes.html). These attributes aim to give the user extra information that may help interpret the annotation, for example, ‘RNA-seq supported only’ or ‘Readthrough transcript’. Figure 2 shows an example of a gene that is affected by a sequence error on the reference genome, which has been corrected with a patch. HG299_PATCH allowed us to annotate the SLC37A4 gene has a coding gene because the single nucleotide insertion that disrupted the coding region was removed.

Since the VEGA genome annotation is incorporated into Ensembl via a gene merge pipeline (5) that runs only ∼3–4 times a year, the annotation shown in Ensembl is at least 6 months older than what is available in the in-house annotation database. To mitigate this delay and to allow the user to view annotation that is updated on a weekly or fortnightly basis, VEGA now has an ‘update’ track for human and mouse. Currently, the update pipeline is run fortnightly but in due course we are aiming to ramp this up to weekly updates and we will also incorporate other species. The advantage of an update track is 2-fold: there will be fewer helpdesk queries about annotation that has already been updated internally but is not yet visible in VEGA, and when a query results in an annotation update, the user and community as a whole, only needs to wait 1–2 weeks to see the update in a browser. The number of genes with updated annotation between releases can be seen on the statistics page (http://vega.sanger.ac.uk/Homo_sapiens/Info/Annotation); in the current human release around 3000 genes have been updated or created, which represents around 6% of the total human gene content.

## NOVEL GENE TRACKS IN VEGA: LOF AND KO

To examine the consequence of single nucleotide variation (SNV) on the structure of transcripts, as part of the study by MacArthur *et al.* (19) to identify all loss of function (LoF) variation in human protein-coding genes, we manually annotated the transcript models associated with 884 putative LoF variants to help users to visualize the SNV consequences. Our main aim with the LoF annotation was to (i) resolve the structure and functional potential of the genes on the reference genome and (ii) predict the potential effect of the variation on the structure and, consequently, functional potential of the transcript. Where possible, transcript models representing the structural effect of the LoF variants were constructed, and these are shown in VEGA. The dbSNP IDs of the relevant SNVs are linked to the transcript models in the database and are searchable. To distinguish the LoF models from the reference HAVANA annotation, they are shown in a separate track in VEGA and their names are prefixed with ‘LOF:’. LoF models for non-sense SNVs and small insertions or deletions (indels) are truncated at the position where the novel stop codon would be in an affected genome. Where premature stop codons are likely to trigger the non-sense-mediated decay (NMD) pathway (20), this is indicated by the use of the NMD biotype for the transcript. As predicting the consequences of splice site disruption can be difficult, particularly for splice donor sites, where there is no additional evidence for novel splice sites, all predictions of the effect of splice junction SNVs on the structure and functional potential of a transcript are conservative. For variations that affect splice acceptor sites, we assume the next confidently identifiable splice acceptor is used. Unless there is transcriptional support for the use of an alternative downstream splice acceptor within the affected exon this equates to a prediction that the exon immediately following the affected splice acceptor is being skipped. The impact of splice donor SNVs is more difficult to predict, as they can have an effect on the splicing of exons upstream as well as downstream of the affected splice site. As such, unless there is transcriptional evidence that covers the disrupted donor site, models representing the effects of splice donor SNVs have not been created.

VEGA’s display of knockout (KO) transcript models is very similar to that of LoF models: KO models show the structural and functional consequences of the removal of target exons in the relevant KO mouse model. The International Knockout Mouse Consortium (IKMC) (https://www.mousephenotype.org) (21) has established a global embryonic stem cell resource containing mutant alleles for more than 18 000 protein-coding genes (http://www.knockoutmouse.org). Such large scale gene targeting was achieved by combining manual target selection and computational design with parallel conditional targeting vector construction and high-throughput gene targeting in C57BL/6 ES cells (22). In collaboration with the European Conditional Mouse Mutagenesis (EUCOMM) (http://www.mousephenotype.org/martsearch_ikmc_project/about/eucomm) and Knockout Mouse Project (KOMP) (http://www.nih.gov/science/models/mouse/knockout/) consortium partners, the HAVANA group was involved in the manual selection of target exons using the Ensembl gene set. This enabled us to select target exons that optimize disruption across all protein-coding alternatively spliced transcripts, while avoiding conserved sequence regions and maintaining conditionality. With the on-going annotation of the mouse genome, we aim to validate target exon selection by assessing alternative splicing using published transcription data. Knockout genes successfully targeted in ES cells will also be represented as a theoretical gene structure to demonstrate the impact the targeted exons have on the coding sequence when they are deleted in the null allele. With these theoretical models, not only are we able to represent the resulting frameshift in the coding region, we can also run our protein analysis pipeline to predict changes in the molecular properties and domain structure of the mutant protein. KO models are available in the VEGA browser as separate tracks for EUCOMM and KOMP knockouts. At the time of writing there are close to 5200 mouse KO genes in VEGA.

## REGION COMPARISON USING THE NOD MOUSE IN VEGA

Since the previous VEGA publication (23), the HAVANA team has annotated or updated 21 regions of the non-obese diabetic (NOD) mouse known to be associated with type 1 diabetes (T1D) (24,25). These candidate regions are referred to as *Idd* regions, an abbreviation of insulin-dependent diabetes. At the same time, the homologous regions in the C57BL/6J mouse (GRCm38 build) were annotated. The NOD mouse spontaneously develops T1D and because it shares many characteristics with the human disease it serves as a model organism for the study of human diabetes and for the evaluation of therapeutic interventions. Characteristics in common include genetic polymorphisms that affect shared pathways, shared antigenic targets and the expression of class II MHC molecules displaying related peptides (25). Comparing the sequences of *Idd* candidate regions between the diabetes-sensitive NOD mouse and the diabetes-resistant C57BL/6J reference mouse should allow the identification of genomic variations putatively associated with diabetes in mice and, by extension, in humans (25).

**Figure 3.**
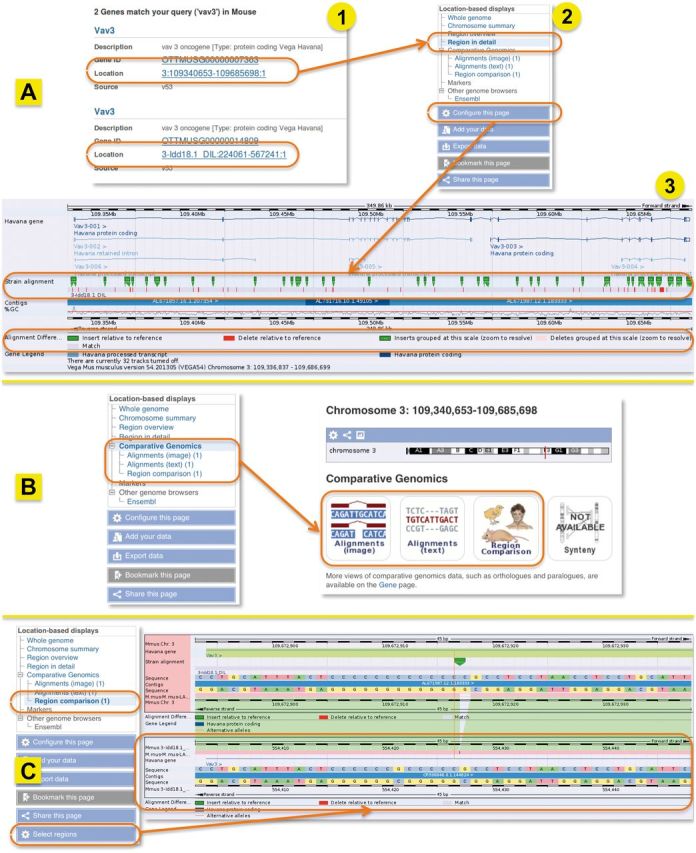
Viewing different haplotypes in VEGA. **(A)** Searching for gene Vav3 in mouse produces two results: one in the C57BL/6J reference mouse and one in the DIL NOD mouse (1). Selecting the location view allows the user to view the ‘Region in detail’. Variation data are available under ‘Configure this page’ (2) and by subsequently selecting ‘Strain alignment’. Insertions and deletions between the two strains can be observed in the strain alignment track, with insertions relative to the reference shown as green blocks and deletions relative to the reference shown as red blocks as detailed in the ‘Alignment Differences’ legend (3). **(B)** VEGA can present alignment data either graphically via the ‘Alignments (image)’ and ‘Region comparison’ sections, or as text via the ‘Alignments (text)’ section. **(C)** To view variations at a nucleotide level between the two strains click on the ‘Region comparison’ panel in the left hand menu and then choose the ‘Select regions’ menu to add the appropriate region. The sequence for the DIL NOD mouse (bottom) can be aligned and visualized against the C57BL/6J reference strain (top). This particular view shows the most 3′ intron of Vav3. An insertion relative to the reference is shown in the middle of the display by a green block. The C57BL/6J mouse clearly has an extra C nucleotide with respect to the DIL NOD mouse. Regions of identity or similarity between the two strains are shaded in green.

Using the ‘Region comparison’ panel in VEGA, completed C57BL/6J mouse annotation can be viewed alongside the NOD mouse annotation, either as text alignments or as graphical alignments (Figure 3). This view allows comparison of the genomic sequence and genes in the candidate loci between diabetes resistant and diabetes sensitive strains. This functionality has been useful for identifying regions of large variation between the two mouse strains, but is only of limited use when looking for small regions of variation such as single nucleotide polymorphisms (SNPs) and short insertions and deletions (indels). We have therefore added a track to VEGA that offers a better way of identifying regions of small difference between the two mouse strains. Figure 3 illustrates the new track, which is made available through the ‘Region comparison’ feature. Example gene Vav3 is a gene that is known to be involved in T1D (26) and has been annotated in both the C57BL/6J mouse and the DIL NOD mouse. The new track, identified as ‘strain alignment’ in the Configuration Menu, clearly shows the indels between the two very similar sequences, even when zoomed-out to display large regions. Where necessary, the track clusters adjacent variations into single visual elements of appropriate size for the displayed scale. We are currently extending this track to view SNPs as well as indels and it can also be used to examine different haplotypic regions.

## COMMUNITY ANNOTATION PORTAL

Since the initial release of the VEGA browser in 2004, the annotation of the human genome has moved from a community annotation project involving sequencing centres collaborating in the Human Genome Project (1) to manual annotation from a single group. However, as manual annotation is a labour intensive and expensive process, only high quality high impact reference genomes have been targeted. To encourage communities built around other organisms to assist with the annotation of their genomes, the HAVANA group runs annotation workshops and provides access to their annotation tools ZMap/otterlace (http://www.sanger.ac.uk/resources/software/anacodeannotools/). This has resulted in a successful collaboration with pig genomic researchers to annotate the porcine immunome (6). More than 1300 immunity-related genes were annotated on swine genome assembly 10.2 and the results can be seen in the VEGA browser. In addition, since the genes are annotated on the same assembly Ensembl uses, Ensembl was able to merge and integrate the annotation into their reference gene build (5). Using the same community annotation approach, we are targeting genes of interest to the rat community in the rat genome and will offer the resulting manual annotation for merging into the Ensembl gene build. We have also implemented the HAVANA update track in the Rat genome contigview so that updated community annotation can be viewed within 2 weeks of annotation release.

## ACCESS TO VEGA, USER STATISTICS AND ACCESSING DATA

VEGA has around 8000 unique visits a month and serves ∼30 000 pages. Users are distributed globally, coming from at least 110 distinct countries, although the majority of users are situated in Europe (UK and Germany), North America (USA) and Asia (China and Japan). The VEGA database can be accessed via a number of cross-referenced collaborative sites, such as Ensembl or specialized databases such as Zfin (27), MGI (28) and CCDS (16). Around 75% of VEGA users enter the site by following links from such sites, the two most popular being Ensembl and NCBI resources. Together with the observation that the most popular VEGA pages are the human, mouse and zebrafish gene and transcript summary pages, this suggests that most users are using VEGA to check on specific aspects of annotation. Nevertheless ∼25% of visits involve views of five or more pages suggesting that that users do explore the other resources VEGA offers.

The gene sets on whole genomes can be accessed in VEGA through the Biomart warehouse system in Ensembl (29), and the data are updated on every VEGA release. Queries to VEGA can be sent directly to developers and annotators using the Helpdesk interface (http://vega.sanger.ac.uk/info/website/help/index.html). The data in VEGA can be downloaded in different ways. First, for regions up to 20 Mbp the annotation can be exported as GFT format from the website. Second, we provide a set of files on our FTP site (ftp://ftp.sanger.ac.uk/pub/Vega). While these are generally limited to FASTA sequence files, other data can be provided on request. Third, for the species incorporated into Ensembl, the databases are available on the public Ensembl MySQL database (ensembldb.ensembl.org) or can be downloaded from the Ensembl FTP site (ftp://ftp.ensembl.org/pub/current_mysql/).

## FUTURE DIRECTION

As the GENCODE project is expanding to mouse to improve its reference annotation, the number of lncRNAs and pseudogenes annotated will increase within VEGA. We have also begun to submit the human lncRNAs annotated within VEGA to the Third Party Annotation database (30) to enable submission to the newly formed federated database RNAcentral (31). This will allow more users to access this highly curated data and allow for it to be integrated into a more comprehensive RNA database. As knowledge concerning the function of lncRNAs in different species improves, we will consider improving our biotype classifications to introduce a more functional lncRNA biotype rather than a positional-based biotype.

Our knowledge of the transcriptional landscape is growing increasingly complex as more next generation analysis becomes available. For example, CAGE (32) and polyAseq (33) allow existing models to be completed and new transcripts to be identified. In combination, the longer full length cDNA reads from new sequencing methods such as PacBio (34) will prove invaluable for annotating the true extent of transcriptional complexity. Functional annotation is also becoming a more proactive process: ribosome profiling (35) can highlight regions of RNA that are translated, while RNA immunoprecipitation technologies identify lncRNAs that interact with specific proteins in the cell. Furthermore, next generation assays of all kinds are being used increasingly to target specific cell types and developmental stages, allowing us to identify the incredible dynamism that exists in the transcriptome. The next challenge for genomic browsers is therefore to condense such information into an informative display, allowing users to interpret what is happening to the expression of their transcript of interest in different tissues.

## FUNDING

National Institutes of Health [5U54HG004555]; the Wellcome Trust [WT098051]; BBSRC rat grant [BB/K009524/1]. Funding for open access charge: Wellcome Trust Sanger Institute.

*Conflict of interest statement*. None declared.
